# Symmetry constraints during the development of anisotropic spinodal patterns

**DOI:** 10.1038/srep20806

**Published:** 2016-02-10

**Authors:** Luis Sánchez-Muñoz, Adolfo del Campo, José F. Fernández

**Affiliations:** 1 Instituto de Cerámica y Vidrio (CSIC), Kelsen 5, 28049 Madrid, Spain

## Abstract

Spinodal decomposition is a phase-separation phenomenon occurring at non-equilibrium conditions. In isotropic materials, it is expected to improve the physical properties, in which modulated structures arise from a single system of spinodal waves. However, in anisotropic materials this process is controversial and not very well understood. Here, we report anisotropic spinodal decomposition patterns in single crystals of K-rich feldspar with macroscopic monoclinic 2/m symmetry. The periodicity of the spinodal waves at ~450 nm produces a blue iridescence, typical of the gemstone moonstone. Stripe patterns in the (010) plane, labyrinthine patterns in the (100) plane, and coexistence of the two patterns in the (110) plane are first resolved by scanning Rayleigh scattering microscopy. Two orthogonal systems of spinodal waves with the same periodicity are derived from the features and orientations of the patterns on the crystal surfaces. The orthogonality of the waves is related to the perpendicularity of the binary axis and the mirror plane. Thus, the spinodal patterns must be controlled by symmetry constraints during phase separation at early stages. Unusual and new properties could be developed in other anisotropic materials by thermal treatment inducing two orthogonal systems of periodic spinodal waves.

Spinodal decomposition is a well-known phase-separation phenomenon of disordered media that produce coherent nanoscale intergrowths, commonly used to improve the physical properties of diverse materials, including metallic alloys^1^,^2^, thermoelectric materials^3^,^4^, magnetic materials^5^, liquid crystals^6^,^7^, polymers^8^,^9^,^10^ and glasses^11^,^12^. Phase separation by spinodal decomposition occurs where a hybrid homogeneous phase can no longer remain metastably, becomes unstable and thus proceeds to unmix. Metastability arises because small changes in energy related to interface formation raise the total free energy of the system. However, where the system becomes unstable, phase separation starts spontaneously by diffusion without forming interfaces, as the interfacial energy barrier is absent. Isotropic materials naturally build up coherent modulated structures at different scales, with typical patterns acquired by the development of a single wave system of long-range spinodal waves^13^,^14^,^15^,^16^,^17^. In elastically isotropic systems like glasses, the amplification of the composition wave is equally probable in all directions, and hence a highly interconnected pattern arising from spherical periodic waves is produced. But in elastically anisotropic crystals, spinodal waves are expected to be amplified mostly in the elastically soft directions. For instance, in 2/m pyroxenes, where the spinodal decomposition phenomenon is well known from kinetic experiments, compositional modulations with diffuse interfaces are parallel to (001) and (100), and fine-scale “island-like” regions are formed before lamellar coarsening develops^18^. Note that many of the functional applications of modern materials nowadays mainly involve non-isotropic crystals. However, knowledge about anisotropic spinodal mechanisms of decomposition is scarce, and consequently, the applications have rarely been explored^19^. Most theoretical predictions in this field are based on external effects designed to create anisotropy within isotropic liquid media^20^.

Feldspars, the most abundant group of minerals in the crust of the Earth and the Moon, do show spinodal decomposition under certain geological conditions^21^,^22^,^23^,^24^,^25^,^26^,^27^,^28^,^29^,^30^,^31^,^32^,^33^,^34^,^35^,^36^. Feldspars crystallize at high temperature with 2/m symmetry in long-range disordered structures forming two solid-solution series known as alkali feldspar (KAlSi_3_O_8_–NaAlSi_3_O_8_) and plagioclase (NaAlSi_3_O_8_–CaAl_2_Si_2_O_8_). During cooling at lower temperatures, phase separation can occur by nucleation and growth or by spinodal decomposition. In the plagioclase series, modulated structures are related to early stages of spinodal decomposition, and it is also the origin of an iridescence phenomenon known as the schiller effect or labradorescence^24^. Iridescence is produced by coherent diffraction of certain wavelengths of white light from periodic domains that are also characterized by the appearance of satellite reflections from crystal superstructures^28^,^32^.

In Na-rich feldspar showing a schiller effect, i.e., moonstone, the relationship between iridescence and unmixing was explained from early X-ray diffraction experiments in 1921 by Kôzu and Endô^37^,^38^. However, detailed correlations between optical effects and the structural and microtextural patterns conflicted with the traditional view of what is a crystal^39^. Two distinct explanations were proposed to clarify the origin of this optical phenomenon, which is very unusual in the solid state. Spencer^40^ suggested scattering and reflection of light by a “schiller plane” along (−601) as the interface between the two exsolved constituents, appearing as lamellae in perthitic intergrowths. Alternatively, Raman and coworkers^41^,^42^ proposed diffusion of light inside crystals of blue Sri Lanka moonstone, where the distribution of the intensity of iridescent light has an elliptical pattern perpendicular to (100). This noncoherent scattering was explained as an optical consequence of pronounced local fluctuations in its chemical composition and refractive indices on a scale smaller than the resolving power of an optical microscope, and thus unrelated to conventional perthitic intergrowths. The optical effect has a maximum intensity when viewed in a particular direction, which is related to the direction of the incidence of light in such manner that the two directions roughly make equal angles with and lie in the same plane as a particular direction within the crystal termed the “schiller axis”^42^.

The Spencer–Raman controversy was apparently solved once the microstructural details could be resolved by transmission electron microscopy (TEM). Preliminary observations revealed the existence of the hypothesized exsolution lamellae in a “white” moonstone. The schiller effect was explained as a direct result of optical interference between Na- and K-rich regions having a wide variation in thickness, although additional incoherent scattering from unmixed regions could also contribute to the diffuseness of the phenomenon^43^. The examination of more specimens demonstrated that the schiller effect can actually be associated with several types of microstructures observed on (001) surfaces, including straight and lenticular films of Na-rich feldspar, as well as wavy and lozenge-shaped morphologies of Na-rich lamellae^31^. Proposed explanations of such microstructural variants were based on two different genetic mechanisms: coherent nucleation and growth as a discontinuous mechanism, and spinodal decomposition as a continuous mechanism, followed by coarsening processes that make it difficult to recognize the early mechanism of exsolution. The spinodal hypothesis was experimentally favored in those cases of Na-rich feldspars where the lamellar spacing vs annealing time1/3 at 600 and 700 °C defined a straight-line relationship, suggesting a diffusion-controlled mechanism^26^.

One of the earliest stages of spinodal decomposition in natural K-rich feldspar has been assumed to occur in specimen Spencer M, a blue moonstone from Sri Lanka consisting of pseudoperiodic wavy lamellae with sharp phase-boundaries. The Na-rich phase shows (010) twinning, and is approximately parallel to [−601]^27^. In this work, we selected other similar moonstone specimens from Sri Lanka having similar chemical composition to Spencer M, but differing in their microstructure. These K-rich feldspars were formed in the granulite facies of metamorphism^44^, involving a geological environment of static high pressure (>3 Kbars), in which spinodal patterns can develop during slow cooling^34^,^35^,^36^. In these geological conditions, homogeneous temperature and pressure in large crustal sections are well known as inferred from mineral paragenesis. Because of the implied dry state of the granulite-facies rocks, the spinodal patterns can be better preserved at early stages, especially where interaction with heated water has not occurred at lower temperatures, and where rapid uplift, well documented in the area^45^, freezes the products of the exsolution process.

## Results

### The iridescence phenomenon

**Figure 1:**
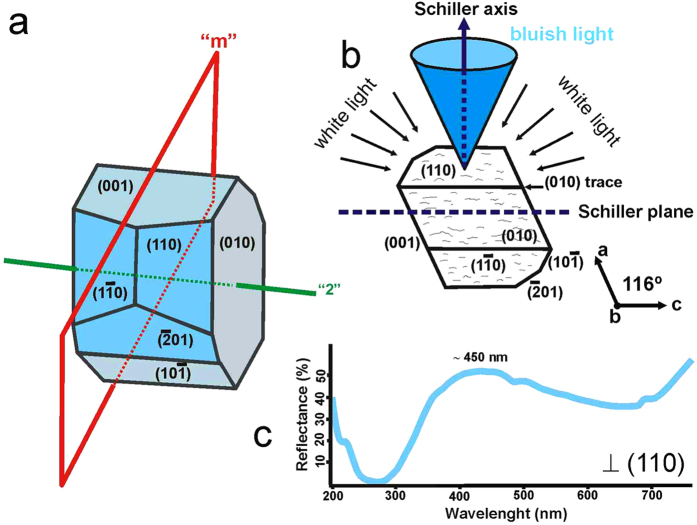
Blue iridescence in monoclinic crystals of moonstone. (**a**) Schematic illustrations of the *C*2/m crystal symmetry and the Miller indices of the main crystal faces. The iridescence phenomenon is mainly observed on (110) and (−201) planes. (**b**) The distribution of the maximum light intensity has an elliptical shape when illuminated with natural white light. (**c**) The diffuse reflectance spectrum, obtained perpendicularly to the (110) plane, shows a maximum consistent with the periodicity of the pattern involved in the optical effect at circa 450 nm.

Figure 1 shows the external crystallographic planes showing the Schiller effect in a sample of blue moonstone, typically on (110), (1–10) and (−201) planes. It also occurs in other directions, but at a much lower intensity^41^. The wavelength of the iridescent light was recorded using UV-visible spectra acquired at various orientations on the transparent and homogeneous moonstone. The maximum reflectance in the blue region of the spectrum indicates a narrow distribution in the periodicity of the modulation, as can be expected at the very early stages of phase separation by spinodal decomposition. Typically, the diffusion halos along the Schiller axis define a wide solid angle, with an elliptical shape^42^. Actually, the Schiller effect is spherical in shape, although with different intensities at different orientations^41^. Rotation of the crystal relative to the direction of incident light causes a decrease in optical effect, i.e., a reduction in the relative intensity of the blue reflectance. The UV-vis spectra of the moonstone effect on (110) and (100) planes (see Supplementary Fig. S1) were collected with an integrating sphere at several angles of rotation of the specimens around the c axis, which is perpendicular to the incident light and in the (110) plane. Starting from the (110) plane, the sample was rotated to reach a maximum reflectivity at near 30°, at which point the light is perpendicular to (100). However, if the light reaches the sample nearly perpendicularly to the (100) plane and the sample is rotated about the [100] direction, the reflectivity does not change significantly.

### Spinodal patterns

At the very early stages of spinodal decomposition, the composition waves are broad and have small amplitude; they thus are difficult to resolve by most conventional techniques. Diffuse scattering in X-ray patterns lacks spatial resolving power to quantify the size distribution and periodicity of the domains. Electron microscopy techniques can resolve the details of the earliest modulated patterns only with difficulty owing to their long wavelength and lack of sufficient chemical contrast for imaging. On the other hand, evolved patterns with sharp interfaces between the discrete phases are relatively well known^46^. Scanning light scattering spectroscopic microscopy^47^,^48^,^49^ could be an alternative technique to visualize the early stages of pattern formation resulting from spinodal decomposition. The scattering of light by matter can be inelastic (i.e., Raman scattering), in cases where frequency shifts are detected between the incident and the scattered light, or elastic (i.e., Rayleigh scattering), where no frequency shift occurs. Raman scattering results from molecular interactions. It was found to be useless to image the resolution of the spinodal patterns, because spectral differences between the two separated phases are essential to create an image. Such differences are absent at the very early stages of spinodal decomposition, as the compositional waves have a low amplitude. However, Rayleigh scattering is structurally specific, and has a high sensitivity to small changes in index of refraction and density. We found it very useful to illustrate the early and intermediate spinodal patterns. The mean index of refraction and density are greater in Na-rich feldspar than in K-feldspar; there is thus a higher intensity of scattered light from the Na-rich component than from the K-rich component of the modulation. Hence, the elastic scattering of laser light was used for image formation in optical microscopy (see Supplementary Fig. S2).

Scanning Rayleigh scattering images were recorded using a modified confocal Raman microscope, with a Nd:YAG laser light having a wavelength of 532 nm. The laser beam is focused perpendicular to the sample surface by a 100× objective (N.A. 0.95). The specimens were mounted on a piezo-driven microscope scanning stage, which allows steps of 3 nm in the X–Y plane and 1.0 nm in the Z plane. Images were formed in areas of several micrometers of the sample surface by step-scanning laser light. We collected the reflected laser light at each point through the same objective, driven along the microscope column and without any filter, using a multimode optical fiber 25 μm in diameter connected to a very sensitive CCD detector that measures the intensity of Rayleigh scattering at every step. The very small diameter of the optical fiber acts as a pinhole and guarantees the confocality of the system. The perpendicularity of the sample’s surface and laser beam is critical to improve the quality of data and the final image. For that reason, the sample was mounted on a tilt-correction device to work with tilts of less than 1 μm in 150 μm of length. We obtained a maximum contrast of light scattering between domains with close chemical composition by moving the sample up and down to maximize the relative intensity of the Rayleigh scattered light. Then the optical system is focused down 200 nm and kept there to avoid any effect of surface roughness. In addition, several powers of laser light were tested in order to determine the best experimental conditions for the resolution of these patterns (see Supplementary Fig. S3). Using available software, we have built images depicting the percentage variation in intensity at each point of the scanned area of the sample surface with respect to the average intensity in the total area. Images were obtained on finely polished (001), (010), (100) and (110) surfaces. At these conditions, it was possible to observe large areas with easy manipulation of the sample at different orientations, having a maximum spatial resolution in the X–Y plane^47^ of up to λ/20. On the basis of variations in the reflectance of sample surfaces, we found two distinct light-scattering statistics in scanning experiments: a bimodal distribution and a Gaussian distribution. Each type of light statistics is correlated with well-defined spinodal patterns, as explained below.

**Figure 2:**
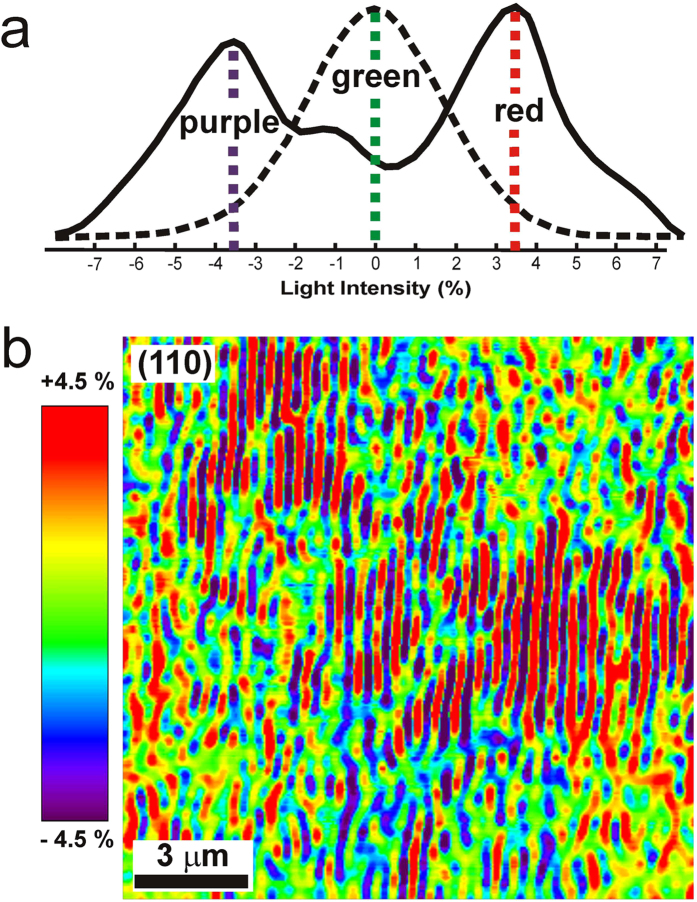
Rayleigh statistics and image formation in polished (110) plane. (**a**) The statistic distribution of the elastic light scattering experiments follows two different distributions. The bimodal distribution (continuous line) with two well defined maxima corresponds with images in which phase separation domains in purple and red color are separated by narrow regions of intermediate composition. The Gaussian distribution (discontinuous line) have a single maximum close to the origin, and it corresponds with images in which the areas of green color are dominant. (**b**) In the (110) plane both statistics coexist side by side and correspond with stripe patterns and labyrinth-island patterns of unmixed domains, respectively. The qualitative color scale in the image varies between +4.5 and −4.5% of relative intensity of the laser scattered light. The density for compositional end-members in alkali feldspars ranges from 2.613 gr/cm^3^ in Na-feldspar to 2.557 in K-feldspar while Refractive Index for the Na-feldspars (1.527, 1.535 and 1.536) are higher than for K-feldspars (1.519, 1.524 and 1.523). Thus, Na-rich domains are visualized in red color, whereas K-rich domains are seen in purple color.

The first type of statistics involves a sharp bimodal distribution of scattered light (continuous line in Fig. 2a). Bimodal scattering results from a **periodic pattern of stripes**. The Na- and K-rich domains have the same thickness and alternate very regularly without sharp phase boundaries, as shown in red and purple in the (110) plane (Fig. 2b) and in the (010) plane (see Supplementary Figs S4a and S4b). Importantly, the striped periodicity observed in the (110) plane is fully compatible with the same value obtained in the (010) plane (see Supplementary Fig. S4c).

The second type of statistics appears only in the (110) and (100) planes and consists of regions with a broad Gaussian distribution in the intensity of the elastically scattered light (discontinuous line in Fig. 2a). Gaussian scattering arises in parts of the crystal where the incipient unmixing cannot be detected (green color in Fig. 2a). These regions with incipient unmixing consist of island and labyrinthine patterns of Na- and K-rich domains of red and purple color, respectively, as shown in Fig. 2b. In the (110) plane, atomic force microscopy (AFM) cannot resolve the spinodal patterns on polished surfaces, and ill-defined microtextures result on etched surfaces (see Supplementary Fig. S5). Etched surfaces improve the image contrast obtained by Rayleigh scattering, but decrease its spatial resolution where a region of small chemical contrast is analyzed (see Supplementary Fig. S6). Similarly, AFM cannot resolve unmixing patterns in polished surfaces of the (010) plane (Supplementary Fig. S7). Hence, we conclude that the (010) plane shows only the stripe pattern, similar to that already resolved in the electron microscopy literature, and the (100) plane shows only the island- and labyrinth-type patterns, which have not yet been described in the literature.

**Figure 3:**
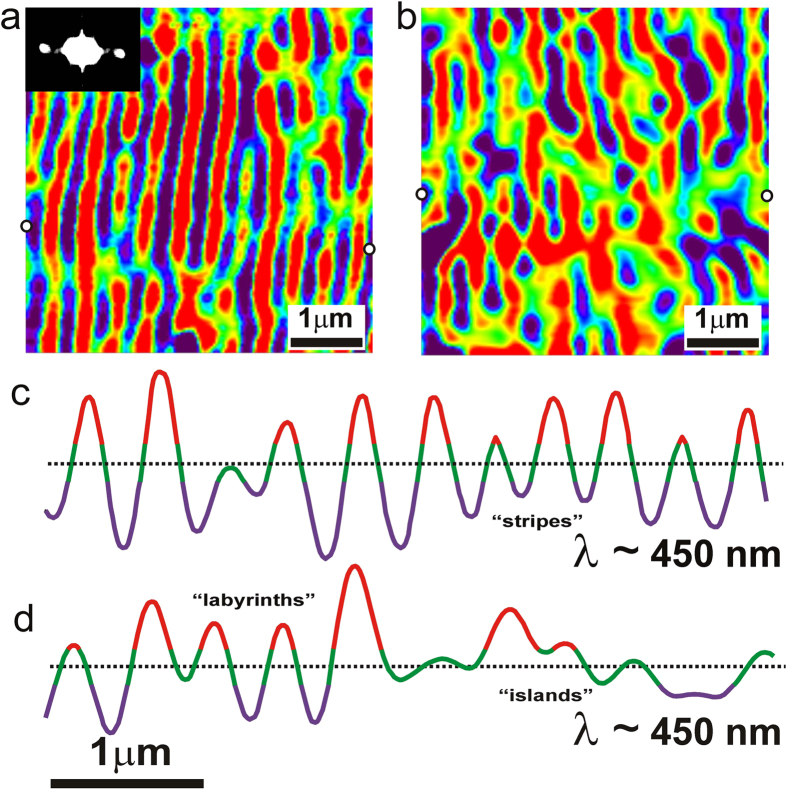
Stripe and island to labyrinthine patterns in anisotropic spinodal patterns in (110) plane. (**a**) The stripe pattern is formed by periodic lamellae of irregular shape but elongated along the [001] direction, with narrow regions of intermediate composition in between the two lamellar types. The splitting of a particular lamella is related to the origin of the other giving rise to a typical Y-shaped configuration. The periodicity of the pattern is recorded in the optical Fourier transform as inset. (**b**) The island and labyrinthine patterns are composed by circular lamellae of one type surrounded by the other type in arcuate shape, with intermediate composition (green color) in between the two types. (**c**) The stripe patterns produce a very characteristic profile of scattered light showing a concentration wave with high amplitude and well defined periodicity at ~450 nm and narrow boundaries about 20–40 nm. (**d**) The labyrinths and islands have the same periodicity but the spinodal waves are more irregular having lower amplitude and very wide regions with intermediate chemical composition. The two white dots at the borders of the images in (**a**,**b**) mark the initial and ending point of the optical profiles. The qualitative color scale in the image is the same than in Fig. 2.

Figure 3a exhibits details of the stripe pattern in the (110) plane. One sees two types of lamellae having similar morphological features, but opposite in chemical composition, as elongate domains along the c axis and approximately parallel to the (100) plane. The two types of domains have the same average thickness, 225 nm, and minor unmixed volumes exist in between. An exceptionally well-defined average periodicity of ~450 nm is determined (see optical Fourier transform as inset in Fig. 3a and line profile of Fig. 3c). The lamellar thickness is exactly half of the observed periodicity. Green regions of unmixed intermediate composition between the red and purple chemical peaks are 20–40 nm in width and signal the resolution limit of our experimental setup. The periodicity of the striped patterns corresponds to the wavelength of blue light detected in the iridescence phenomenon. Periodicity is only disrupted by sharp splitting of one component and ending of the other component, resulting in a typical Y-shaped configuration. Note that no sharp boundaries are detected in the patterns, which also is a typical feature of spinodal patterns.

Figure 3b shows the island- and labyrinth-type nanopatterns in the (110) plane. These consist of dot-shaped domains surrounded by other domains with arcuate or circular shape, i.e., one component turns around the other while maintaining constant the lamellar width and periodicity of approximately 450 nm (Fig. 3d). This configuration is actually a rotation of the stripe pattern and is associated with a larger development of regions of green color (i.e., close to the unexsolved feldspar) between the red and purple components. The pattern rotation explains the broad solid angle and the elliptical shape of the diffusion halo of iridescent light almost perpendicular to (100). In addition, the amplitude of the spinodal waves in the rotated patterns is lower than in the striped pattern, explaining the low intensity of scattered light having spherical distribution. More gradual changes in concentration between the two exsolved components exist here than in the striped pattern, explaining also the difficulties in resolution encountered with most conventional techniques.

It is important to note that the pattern of periodic stripes is very different from the typical non-periodic host–guest microtextural relationship found where exsolution of alkali feldspars occurs by nucleation and growth, in which case sharp and straight phase boundaries exist along (−601) planes (31). We have also found that elastic and inelastic scattering have very different capabilities in pattern resolution. Spinodal patterns cannot be resolved by Raman imaging, as only one type of spectrum was obtained all over the surfaces (see Supplementary Fig. S8). In contrast, Raman scattering was very useful to visualize patterns of nucleation and growth in exsolution, as two spectra can be distinguished, one for the Na-rich phase, the other for the K-rich phase, both used to build up the Raman images (see Supplementary Fig. S9).

## Discussion

**Figure 4:**
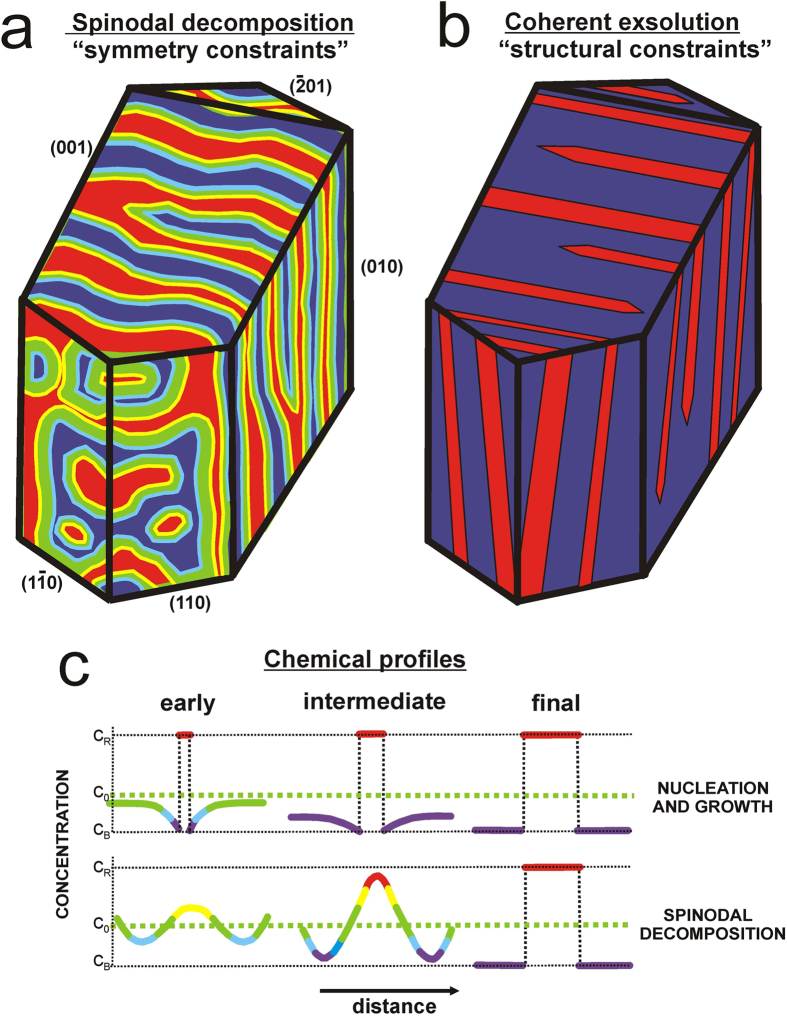
Crystal Symmetry vs. Atomic Structure as constraints for phase separation. (**a**) 3D idealization of the spinodal patterns in different crystallographic planes induced from experimental data obtained by means of Rayleigh scattering spectroscopic microscopy. Regions with intermediate composition between Na- and K-rich lamellae are shown in yellow, green and red color. Symmetry constrains are responsible for the observation of different periodic patterns in each plane. (**b**) 3D idealization of nucleation and growth exsolution patterns as coherent intergrowths without intermediate composition between non-periodic lamellae of Na-rich and K-rick compositions, with well defined crystallographic orientations along the (−601) plane because of local structural constraints. (**c**) Relationship of the 3D models with the schematic evolution concentration profiles similar to those represented by Cahn (1968)^16^. C0, CR, and CB are the original, Na-rich phase and K-rich phase, respectively. If nucleation and growth occurs, phases with very different chemical composition exist since the beginning, but if spinodal decomposition takes place two composition waves related to macroscopic symmetry element of the crystal will develop patterns like those idealized in (**a**).

Regular patterns arising from phase separation can be produced by competing interactions, in addition to nucleation and growth, and spinodal decomposition. The last two mechanisms produce very different nanopatterns (Fig. 4a,b) and chemical profiles (Fig. 4c) at early and intermediate stages. An example of nucleation and growth can be found in Supplementary Fig. S9, where the pattern is not periodic, the phase boundaries are sharp, the width of the two lamellae are different, and they have a wide size-distribution. Nucleation involves a discrete interface at the onset of phase separation. In this case, the structural arrangement of the atoms controls the orientation of the exsolved phases from the very beginning of the phase-separation process, and phases grow parallel to and along the Murchison plane, to minimize the lattice misfit between the two exsolving phases (Fig. 4b). Thus, the nucleation and growth mechanism^22^,^30^,^31^,^46^ radically differs from those of spinodal decomposition and competing interactions, and it cannot explain the features found in the blue moonstone specimens. These patterns are formed in K-rich feldspars with very similar composition to those with spinodal patterns but formed in volcanic environment, i.e., at low pressure.

When modulated phases in macroscopic and mesoscopic patterns are stabilized by competing interactions, patterns consist of stripe and bubble domains in a wide variety of physical-chemical systems in equilibrium, where domains represent modulations in some order parameter^50^. This phenomenon occurs typically in system with a fast kinetic response like liquids and polymers. The experimental patterns are periodic or pseudo-periodic, but do not have a “wave” shape because phase boundaries are normally sharp, although in some cases phase separation can be modeled by means of an order parameter following a sinusoidal wave, as in polymers^51^. These particular features are not found in alkali feldspar crystals, as they have very slow kinetics occurring on the geological time-scale^35^; also, the modulated structures can be described with a composition wave having a well-defined periodicity, which in addition is extremely homogeneous at the centimeter scale. Furthermore, the two phases have the same width but a different amplitude, and are separated by diffuse boundaries, i.e., the modulation has a wave shape (see Fig. 3). The shape and configuration of the modulation wave seems indicative of a spinodal decomposition phenomenon.

To produce such a spinodal pattern with a wavelength of ~450 nm, a temperature circa 550 °C is induced, close but below the coherent spinodal temperature and higher than the monoclinic-triclinic transition (450–500 °C). At this temperature, where the energy terms are close to being balanced, the spinodal wavelength with small amplitude can be very large. The rate of decomposition must be extremely low because of the long paths of diffusion involved. Thus it is not possible to represent their temporal evolution in the Cahn plot^52^, at the laboratory scale because single-crystals must be used at high pressure and high temperature during month or years for the K-rich compositions of these feldspars. In addition, thermal experiments at low pressure will involve nucleation and growth as shown in Supplementary Fig. S9. However, the simulations of the lamellar morphology by numerical modeling are successful in view of the very slow cooling rate and in cases where the interfacial energy is taken to be anisotropic. In contrast, spherical precipitates or irregular curved co-continuous shapes are obtained when the interfacial energy is taken to be isotropic^35^. Note that the symmetry of the phase is considered to be irrelevant for pattern formation. The orientation of the spinodal waves is consistent with the need to minimize the elastic strain energy, and thus corresponds to elastically soft orientations in the crystal. McConnell (1971) has suggested that the fluctuation waves of spinodal decomposition *may have* a transverse component because of the *C*2/m point-group symmetry of feldspars, which should be manifested into the spinodal patterns^53^. This aspect has not been observed in feldspars. We have serious doubt on the validity and specific nature of the spinodal hypothesis in non-isotropic crystals and its relevance to the iridescence phenomenon having a spherical distribution of scattered light. Moreover, transverse spinodal waves have not been described yet in other anisotropic materials in connection with macroscopic symmetry.

The modulation of the stripe pattern is fully consistent with a single spinodal wave. The direction of propagation and the amplitude vector are approximately parallel to the mirror plane and perpendicular to the binary axis of the overall 2/m symmetry of the crystal. However, the island- and labyrinth-type patterns suggest an incipient stage of chemical segregation that cannot be explained with the orientation of the composition wave involved in the stripe pattern. Hence, a second wave system has been induced orthogonal to the first one, but with the same periodicity as the first wave. The second wave has a direction of propagation perpendicular to the mirror plane, and the amplitude vector is perpendicular to the binary axis. Islands and labyrinths in (100) are easily explained as the effects of chemical peaks of this second system of waves. The lower amplitude of concentrations of the second system, compared with the first, can be explained by a local structural resistance to progress the exsolution process, because in this case the orientation of the two phases maximizes the mismatch of their lattice^54^. It could also be related to anisotropic Na-K interdiffusion in the alkali sublattice^55^, as is found in other silicates^18^. The mutual orientations of the spinodal patterns resolved by scanning Rayleigh scattering spectroscopic microscopy indicate that the direction of propagation and the amplitude vector of the second wave system are perpendicular to the first one, just as the binary axis and the mirror plane also obey the 2/m symmetry. We contend that long-range spinodal waves (as far-from-equilibrium effects) are independent of structural detail at the local scale but are constrained by the overall monoclinic 2/m symmetry of the crystal, as anticipated by McConnell in 1971^53^. Diffuse phase-boundaries formed during the early stages of exsolution become distinct and sharp at the end of the exsolution process, as in Spencer M^27^.

Finally, the existence of spinodal decomposition in disordered anisotropic media, with multiple composition waves following the rules of symmetry, opens the possibility to develop and explore unusual and new properties, particularly in those materials having interactions with electromagnetic waves.

## Methods

### Samples preparation

We used two specimens from Sri Lanka in this study, a blue moonstone and a white moonstone. In addition, a specimen of alkali feldspar from Rabb Park (New Mexico, USA) was studied along (001) and (010) planes in polished and etched surfaces, in order to compare the resolution capabilities of Raman and Rayleigh imaging techniques. The blue moonstone and the Rabb Park specimen have very similar chemical composition. The specimens were cut along the (001), (010), (010) and (110) planes using as guides the well-known orientation of the (001) and (010) cleavage planes. Surfaces were then polished using 1 μm diamond paste and cleaned with acetone and ethanol. Chemical etching experiments were carried out using HF vapor (50% in water) during 30 seconds to improve the visualization of phase-separation patterns.

### Electron micro-probe analysis

Quantitative chemical analysis of the specimen used in the figures for Si, Al, Na, K and Ca were performed by electron-microprobe analysis (EMPA). We employed a point-counting technique with a JEOL Superprobe JXA-8900M operated at 15 kV and 25 nA. A 5 μm square electron beam was used to minimize Na loss and to obtain bulk-chemical analysis (Table 1). It is not possible to get a quantitative chemical composition of the two phases by EMPA methods owing to the scale of the modulation. The chemical compositions can be expressed as Or_x_Ab_y_An_z_ (x + y + z = 100) with Or, Ab and An expressing the molar content of KAlSi_3_O_8_, NaAlSi_3_O_8_ and CaAl_2_Si_2_O_8_ components in the solid solution, respectively, as an average result of five analyses per specimen.

### UV-visible spectroscopy

Experiments were performed on a PerkinElmer Lambda 950 spectrophotometer (Shelton, Connecticut, USA) on the (110) plane. The sample was placed within the integrating sphere right in the center of it by a homemade device that allowed us to rotate the sample. Initially, the sample was placed with the (110) plane perpendicular to the incident light beam. Then, we took spectra at different angles by rotating the sample around an axis perpendicular to the incident light and parallel to the (110) sample plane. Moreover, we recorded different spectra by rotating the sample around an axis perpendicular to the (110) plane and maintaining fixed the angle between the incident beam of light and the (110) plane.

### Scanning Raman/Rayleigh light scattering spectroscopic microscopy

Experiments were recorded using a modified confocal Raman microscope. The equipment (Witec Alpha300 RAS) consists of a Zeiss microscope with a Nd:YAG laser light of wavelength 532 nm. The laser radiation is transmitted through a 3.5 μm diameter core that is a wavelength-specific, single-mode, polarization-preserving fiber. The laser beam is collimated via an achromatic lens and passes a holographic band-pass filter, and it is focused perpendicular to the sample surface by a 100x objective lens (NA = 0.95). The specimens were mounted on a piezo-driven microscope scanning stage, which allows steps of 3 nm in the X–Y plane and 1.0 nm in the Z plane. The microscope base is equipped with an active vibration-isolation system, active over 0.7–1000 Hz. To build up an image, an area typically 8 × 8 μm of the sample surface was scanned with the laser on a grid having 240 × 240 steps. The reflected laser light at each point was collected through the same objective using a multimode optical fiber 25 μm in diameter to a very sensitive CCD detector, normally with an integration time of 0.02 of a second. Images were taken at different laser-polarization directions and at different values of laser power, between 0.5 and 500 μm, as measured with a Thorlabs optical power meter. Raman scattered light is focused into a multimode optical 25 μm fiber that serves as the entrance slit for the spectrometer. Detection is made by a back-illuminated 1024 × 127 pixel CCD camera operating at –60 °C. We used a grating of 1800 lines per millimeter grating, resulting in spectral resolution down to 0.02 cm^–1^. With the Witec Project Plus software, we have built images depicting the percentage variation of the Rayleigh scattered light intensity at each point of the scanned area of the sample surface with respect to the average intensity in the total area.

### Atomic force microscopy

The experiments were performed using the same confocal Raman microscope, Witec alpha-300RA (Ulm, Germany) coupled with an apparatus for atomic force microscopy. The sample was imaged in AFM tapping Mode using ArrowFM cantilevers (Nanoworld, Germany), with a resonance frequency in the range of 70–90 kHz and a damping of r = 50%, recording both topography and phase images simultaneously. Images were recorded on both polished and chemically etched surfaces.

### Field emission scanning electron microscopy

The observations by field-emission scanning electron microscopy, FESEM, were obtained using the same surface studied by the other techniques. Images were obtained with secondary electrons using a Hitachi S-4700 (Tokyo, Japan) equipment, with a spatial resolution lower than 50 nm.

## Additional Information

**How to cite this article**: L. Sánchez-Muñoz. *et al.* Symmetry constraints during the development of anisotropic spinodal patterns. *Sci. Rep.* **6**, 20806; doi: 10.1038/srep20806 (2016).

## Supplementary Material

Supplementary Information

## Figures and Tables

**Table 1:** EPMA bulk chemical compositions.

Samples	SiO_2_	Al_2_O_3_	Na_2_O	CaO	K_2_O	Or_x_Ab_y_An_z_
Sri Lanka blue moonstone	65.18	18.62	4.71	0.22	10.01	Or_57.7_Ab_41.2_An_1.1_
Rabb Park feldspar (Figure S9)	65.98	19.21	4.44	0.15	10.53	Or_60.5_Ab_38.8_An_0.7_

## References

[b1] Kato M., Mori T. & Schwartz L. H. Hardening by spinodal modulated structure. Acta Metall. 28, 285–290 (1980).

[b2] Kim M. U. *et al.* Applications of spinodal decomposition to produce metallic glass matrix composite with simultaneous improvement of strength and plasticity. Met. Mater. Int. 15, 193–196 (2009).

[b3] Wu H. J. *et al.* Broad temperature plateau for thermoelectric figure of merit ZT > 2 in phase-separated PbTe0.7S0.3. Nature Comm. 5, 4515 (2014). 10.1038/ncomms5515 25072798

[b4] Androulakis J. *et al.* Spinodal decomposition and nucleation and growth as a means to bulk nanostructured thermoelectrics: Enhanced performance in Pb_1−x_Sn_x_Te-PbS. J. Am. Chem. Soc. 129, 9780–9788 (2007). 17629270 10.1021/ja071875h

[b5] Sato K., Katayama-Yoshida H. & Dederichs P. H. High Curie temperature and nano-scale spinodal decomposition phase in dilute magnetic semiconductors. Jpn. J. Appl. Phys. 44, L948–L951 (2005).

[b6] Nagaya T. & Gilli J. M. Experimental study of coarsening dynamics of the zigzag wall in a nematic liquid crystal with negative dielectric anisotropy. Phys. Rev. E 65, 051708 (2002). 10.1103/PhysRevE.65.051708 12059578

[b7] Bukusoglu E., Pal S. K., de Pablo J. J. & Abbott N. L. Colloid-in-liquid crystal gels formed via spinodal decomposition. Soft Matter 10, 1602–1610 (2014). 24651134 10.1039/c3sm51877a PMC4212980

[b8] Nisato G., Ermi B. D., Douglas J. F. & Kari A. Excitation of surface deformation modes of a phase-separating polymer blend on a patterned substrate. Macromolecules 32, 2356–2364 (1999).

[b9] Sehgal A., Ferreiro V., Douglas J. F., Amis E. J. & Karim A. Pattern-directed dewetting of ultrathin polymer films. Langmuir 18, 7041–7048 (2002).

[b10] Julthongpiput D., Zhang W., Douglas J. F., Karim A. & Fasolka M. J. Transition from Pattern-directed to isotropic dewetting of polymer films on chemically micropatterned surfaces with differential surface energy contrast. Soft Matter 3, 613–61 (2007). 10.1039/b608630f 32900025

[b11] Suzuki M. & Tanaka T. Thermodynamic prediction of spinodal decomposition in multi-component silicate glass for design of functional porous glass materials. High Temp. Mater. Processes 31, 323–328 (2012).

[b12] Madhar N. A. & Varma K. B. R. Spinodal decomposition in tellurite-based glasses induced by excimer laser irradiation. J. Am. Ceram. Soc. 92, 2609–2615 (2009).

[b13] Hillert M. A Solid Solution Model for Inhomogeneous systems. Acta Metall. 9, 525–536 (1961).

[b14] Cahn J. W. & Hilliard J. E., Free energy of a nonuniform system. I. Interfacial free energy. J. Chem. Phys. 28, 258–267 (1958).

[b15] Cahn J. W. Phase separation by spinodal decomposition in isotropic systems. J. Chem. Phys. 42, 1, 93–99 (1965).

[b16] Cahn J. W. Spinodal decomposition. Trans. Metall. Soc. AIME 242, 166–180 (1968).

[b17] Binder K. & Fratzl P. Spinodal decomposition in *Phase transformations in materials* (ed. Kostorz G.) 409–480 (WILEY-VCH Verlag GmbH, 2001).

[b18] Weinbruch S., Styrsa V. & Müller W. F. Exsolution and coarsening in iron-free clinopyroxene during isothermal annealing. Geochim. Cosmochim. Acta 67, 5071–5082 (2003).

[b19] Higgins A. M. & Jones R. A. L. Anisotropic spinodal dewetting as a route to self-assembly of patterned surfaces. Nature 404, 476–478 (2000). 10761910 10.1038/35006597

[b20] Essery R. L. & Ball R. C. Anisotropic spinodal decomposition. Europhys. Lett. 16, 379–384 (1991).

[b21] Christie O. H. J. Spinodal precipitation in silicates. I. Introductory application to exsolution in feldspar. Lithos 1, 187–192 (1968).

[b22] Yund R. A. & McCallister R. H. Kinetics and mechanism of exsolution. Chem. Geol. 6, 5–30 (1970).

[b23] Owen D. C. & McConnell J. D. C. Spinodal behaviour in an alkali feldspar. Nature 230, 118–119 (1971).

[b24] Nissen H. U., Champness P. E., Cliff G. & Lorimer G. W. Chemical evidence for exsolution in a labradorite. Nature 245, 135–137 (1973).

[b25] Owen D. C. & McConnell J. D. C. Spinodal unmixing in alkali feldspar In The Feldspars (eds. MacKenzie W. S. & Zussman J.) 424–439 (Manchester Univ. Press, 1974).

[b26] Yund R. A., McLaren A. C. & Hobbs B. E. Coarsening kinetics of the exsolution microstructure in alkali feldspars. Contrib. Mineral. Petrol. 48, 45–55 (1974).

[b27] Lorimer G. W. & Champness P. E. Origin of the phase distribution in two perthitic alkali feldspars. Phil. Mag. 28, 1391–1403 (1973).

[b28] Wenk H.-R. Ordering of the intermediate plagioclase structure during heating. Am. Mineral. 63, 132–135 (1978)

[b29] Ribbe P. H. Exsolution textures in ternary and plagioclase feldspars; interference colors. Rev. Mineral. 2, 241–270 (1983).

[b30] Parsons I. & Brown W. L. Feldspars and the thermal history of igneous rocks In Feldspars and Feldspathoids (ed. Brown W. L.) 317–371 (D. Reidel Publishing Company, 1984).

[b31] Smith J. V. & Brown W. L. In Feldspar minerals: I. Crystal structure, physical, chemical and microtextural properties (Springer Verlag, 1988).

[b32] Carpenter M. A. Subsolidus phase relations of the plagioclase feldspars solid solutions In Feldspars and their reactions (eds. Parsons I.) 221–269 (Kluwer Academic Press, 1994).

[b33] McConnell J. D. C. The origin and characteristics of the incommensurate structures in the plagioclase feldspars. Can. Mineral. 46, 1389–1400 (2008).

[b34] Petrishcheva E. & Abart R. Exsolution by spinodal decomposition I: Evolution equation for binary mineral solutions with anisotropic interfacial energy. Am. J. Sci. 309, 431–449 (2009).

[b35] Abart R., Petrishcheva E., Wirth R. & Rhede D. Exsolution by spinodal decomposition II. Perthite formation during slow cooling of anatexites from Ngoronghoro, Tanzania. Am. J. Sci. 309, 450–475 (2009).

[b36] Tajcmanova L., Abart R., Wirth R., Habler G. & Rhede D. Intracrystalline microstructures in alkali feldspars from fluid-deficient felsic granulites: a mineral chemical and TEM study. Contrib. Mineral. Petrol. 164, 715–729 (2012).

[b37] Kôzu S. & Endô Y.. X-ray analysis of adularia and moonstone and the influence of temperature on the atomic arrangement of these minerals. Science Reports, Tokyo Imperial University, Ser. 3 1, 1–17 (1921).

[b38] Tutton A. E. H. The structure of adularia and moonstone. Nature 108, 352–353 (1921).

[b39] Laves F. The growing field of mineral structures In Fifty years of X-ray diffraction (ed. Ewald P. P.) 174–189 (IUCr N.V.A. Oosthoek’s Uitgevermaatschappij, 1962).

[b40] Spencer E. Schiller in moonstone. Current Science 4, 95–96 (1951).

[b41] Raman C. V., Jayaraman A. & Srinivasan T. K. The structure and optical behaviour of the Ceylon moonstones. Proc. Indian Acad. Sci. 32A, 123–140 (1950).

[b42] Raman C. V. More about the iridescent feldspars. Current Science 20, 85–87 (1951).

[b43] Fleet S. G. & Ribbe P. H. An electron-microscope investigation of a moonstone. Phil. Mag. 8, 1179–1187 (1963).

[b44] Harder H. Moonstone mining in Sri Lanka: new aspects. J. Gemm. 23, 1, 27–35 (1992).

[b45] Tamashiro I., Santhosh M., Sajeev K., Morimoto T. & Tsunogae T. Multistage orthopyroxene formation in ultrahigh-temperature granulites of Ganguvarpatti, southern India: implications for complex metamorphic evolution during Gondwana assembly. Jour. Mineral. Petrol Sci. 99, 279–297 (2004).

[b46] Parsons I. & Lee M. Mutual replacement reactions in alkali feldspars I: microtextures and mechanisms. Contrib. Mineral. Petrol. 157, 641–661 (2009).

[b47] Boustany N. N., Boppart S. A. & Backman V. Microscopic imaging and spectroscopy with scattered light. Annu. Rev. Biomed. Eng. 12, 285–314 (2010). 20617940 10.1146/annurev-bioeng-061008-124811 PMC3357207

[b48] Backman V. *et al.* Detection of preinvasive cancer cells. Nature 406, 35–36 (2000). 10894529 10.1038/35017638

[b49] Cherkezyan L. *et al.* Interferometric spectroscopy of scattered light can quantify the statistics of subdiffractional refractive-index fluctuations. Phys. Rev. Lett. 111, 033903 (2013). 23909326 10.1103/PhysRevLett.111.033903 PMC4123763

[b50] Seul M. & Andelman D. Domain shapes and patterns: The phenomenology of modulated phases. Science 267, 476–483 (1995). 17788780 10.1126/science.267.5197.476

[b51] Marko J. F. & Witten T. A. Phase separation in a grafted polymer layer. Phys. Rev. Lett. 66, 1541–1544 (1991). 10043235 10.1103/PhysRevLett.66.1541

[b52] Binder K. Dynamics of phase separation and critical phenomena in polymer mixtures. Colloid Polymer Sci. 265, 273–288 (1987).

[b53] McConnell J. D. C. Electron-optical study of phase transformations. Mineral. Mag. 38, 1–20 (1971).

[b54] Willaime C. & Brown W. L. A coherent elastic model for the determination of exsolution boundaries: Application to the feldspars. Acta Cryst. A30, 316–311 (1974).

[b55] Schäffer A.-K., Jäpel T., Zaefferer S., Abart R. & Rhede D. Lattice strain across Na–K interdiffusion fronts in alkali feldspar: an electron back-scatter diffraction study. Phys. Chem. Minerals 41, 795–804 (2014). 10.1007/s00269-014-0692-y PMC4509562 26213440

