# Supplementary material for: Symmetry constraints during the development of anisotropic spinodal patterns
Source: Sci Rep. 2016 Feb 10;6:20806. doi: 10.1038/srep20806 (PMC4748404; doi:10.1038/srep20806)

Symmetry constraints during the development of anisotropic spinodal patterns

Luis Sánchez-Muñoz*, Adolfo del Campo, and José F. Fernández
Instituto de Cerámica y Vidrio (CSIC), Kelsen 5, 28049 Madrid, Spain
*Corresponding author: lsm@icv.csic.es

Figure S1.- UV-vis spectroscopy and blue iridescence. The UV-vis spectrum of the moonstone effect on (110) and (100) plane, with the trace of (010) plane in vertical position, was collected with an integrating sphere at several rotation angles of the specimens along the *c*-axis. Measurements at several rotation angles are presented with the value of the reflectance at 400 nm in brackets. In a) spectra are recorded staring from the (110) plane to reach the (100) plane, whereas in b) rotation of the specimen continues going apart from (100). c) The blue moonstone effect is shown with two different orientations of incident light (note the projection of the shadow of the tablet containing the sample on the flat surface).

Figure S2.- Experimental setup: Raman and Rayleigh scattering spectroscopic microscopy. Four elements are needed, including a laser light illumination, an optical microscope on an active vibration isolation system, a diffraction grating with a CCD detector, and a computer to control measurements and detectors, the Z- and X-Y stages and data acquisition and analysis.

Figure S3.- Variation of the signal distribution with the power laser light. It was found that the optical resolution and the image contrast (depending on the relative intensity of scattered light) change with the power of the used laser light. The effect of the laser power was tested in areas showing island to labyrinthine patterns. a) The figure shows five images of similar areas using five different laser powers, i.e., 0.40 μW, 2.13 μW, 11.3 μW, 47 μW and 483 μW. The maximum resolution increases up to a value close to 12 μW and then no large benefits are obtained for much higher powers. b) The statistics of light scattering recorded by the number of pixels with a certain relative intensity for the five previous images correlates well with the observed resolution in the Rayleigh images, that is, a wider spectral statistic is related to a better pattern resolution.

Figure S4.- Rayleigh scattering scanning microscopy. Raw Rayleigh image on the (010) plane in a) and processed image in rainbow color scale in b) with the same scale of relative intensity of scattered light than in images on the (110) plane (Fig. 2b). c) Profile of relative intensity across the line drawn in Figure S3b. Note that the periodicity in (010) plane is very similar to that in (110) but with a much lower relative intensity from elastic light contrast, due to the lower refraction index difference between Na-rich and K-rich regions in the (010) plane that in the (110) plane. Thus, of striped patterns resolved in the (110) and (010) planes correspond with the same intergrowth, but a much lower resolution image is obtained in the (010) plane.

Figure S5.- AFM images and profiles on the polished and etched surfaces in the (110) plane. To avoid any influence of topography on the scanning Rayleigh scattering images, the sample surface was finely polished achieving a roughness less than ten nanometers. a) 8x8 μm topography image in AFM tapping mode on a polished surface. b) Topography 4x4 μm AFM tapping AFM image on etched surface. c) Lines profiles marked in white color in a) and b) recorded by AFM to show the effect of etch on the surface. AFM is useless to resolve spinodal patterns in the polished surface, only polishing scratches and a very fine roughness unrelated to the spinodal pattern are seen. Etched surfaces develop similar patterns to those of natural polished surfaces, reminiscent of spinodal decomposition. However, the resolution and definition of the obtained image is much lower that the obtained by Rayleigh imaging on the polished surface.

Figure S6.- Scanning Rayleigh scattering microscopy images and profiles in the polished and etched (110) surface. a) 8x8 μm image on the polished surface using the (110) plane; b) 4x4 μm image on the etched surface using the same plane. c) Lines profiles marked in white color in a) and b) to show the change of light intensity (in CCD counts) with distance and the effect of etch on the spinodal microtexture. The effect of etching on the (110) surface involves an increase of the signal contrast between the two types of domains but a decrease in the spatial resolution, as defects are created as a consequence of the chemical attack.

Figure S7.- AFM images and profiles on the polished surfaces in the (110) plane. As in Figure S4, the sample surface was finely polished achieving a roughness less than ten nanometers to avoid any influence of topography on the images. a) 15x15 μm topography image in AFM tapping mode on the polished surface; b) Line profile marked in white color in a). Again, the AFM method is useless to resolve spinodal patterns in the polished surface.

Figure S8. Raman spectra of the blue moonstone specimen in the (010) plane. a) Spectra were obtained in different experimental condition to resolve the distribution of the two types of domains observed by Rayleigh scattering, including the polarization of the Raman signal at 0º and 90º. However, a single Raman spectrum throughout the surface was obtained. b) The Raman peak at ~513 cm^{-1} of feldspar specimens with spinodal decomposition or nucleation and growth are different. In the first case, a single peak with Gaussian shape was recorded, whereas in the second case two distinct Raman shifts from the K- and Na-rich phases were obtained (see deconvolution of this signal in Figure S9e). Note that K- and Na-feldspar end-members show this band at 514.5 and 510.1 cm-1 respectively (*). Thus, the inability to resolve the pattern of K- and Na-rich lamellas by Raman microscopy was due to the very close chemical composition of the two phases in the early unmixing stages instead of a lack of spatial resolution caused by the small size of the lamellas.

Figure S9.- Exsolution pattern by nucleation and growth. Because of the lack of spatial resolution by scanning Raman microscopy we selected another feldspar specimen from Rabb Park (NM, USA) with very similar chemical composition to that of the blue moonstone from Sri Lanka. In this case the exsolution microtexture was resolved by several techniques, having the typical features of phase separation by nucleation and growth. a) Topography AFM image in tapping mode, with a higher resolution image as inset. b) FESEM micrograph using secondary electrons. c) Raman spectra on (001) plane at the same orientation than in the images. d) Detail of the more intense Raman shift, with * in c), and the positions for the two components. e) Statistical distribution throughout the image of the Raman shift for the more intense Raman band, showing the two components in red and purple of the exsolution pattern. f) Raman image of the phase separation microtexture on (001) plane. McKeown, D.A. Raman spectroscopy and vibrational analyses of albite: From 25 ºC through the melting temperature. *Am. Mineral.* **90**. 1506-1517 (2005).

Supplementary Figure 1

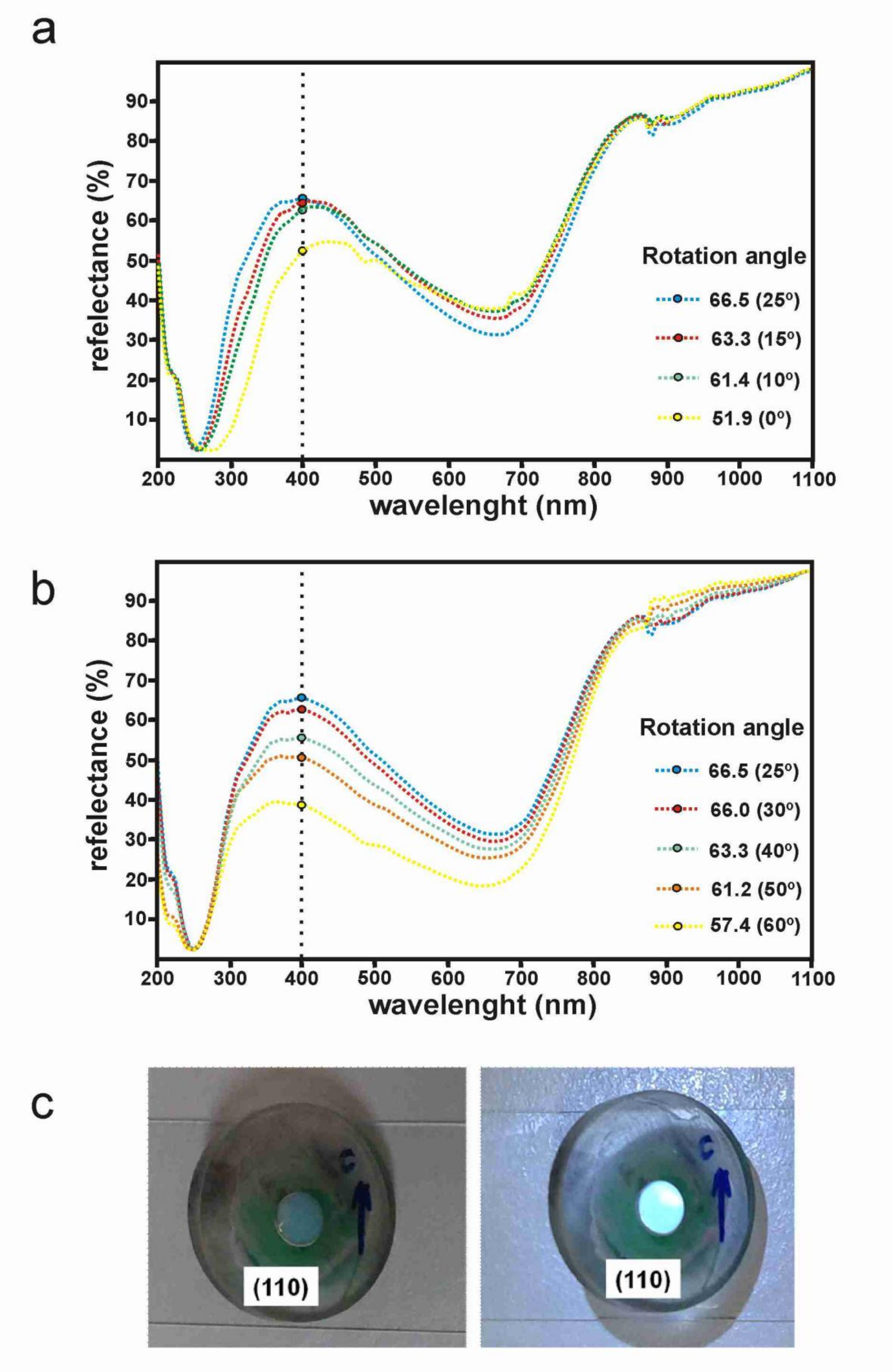

Supplementary Figure 2

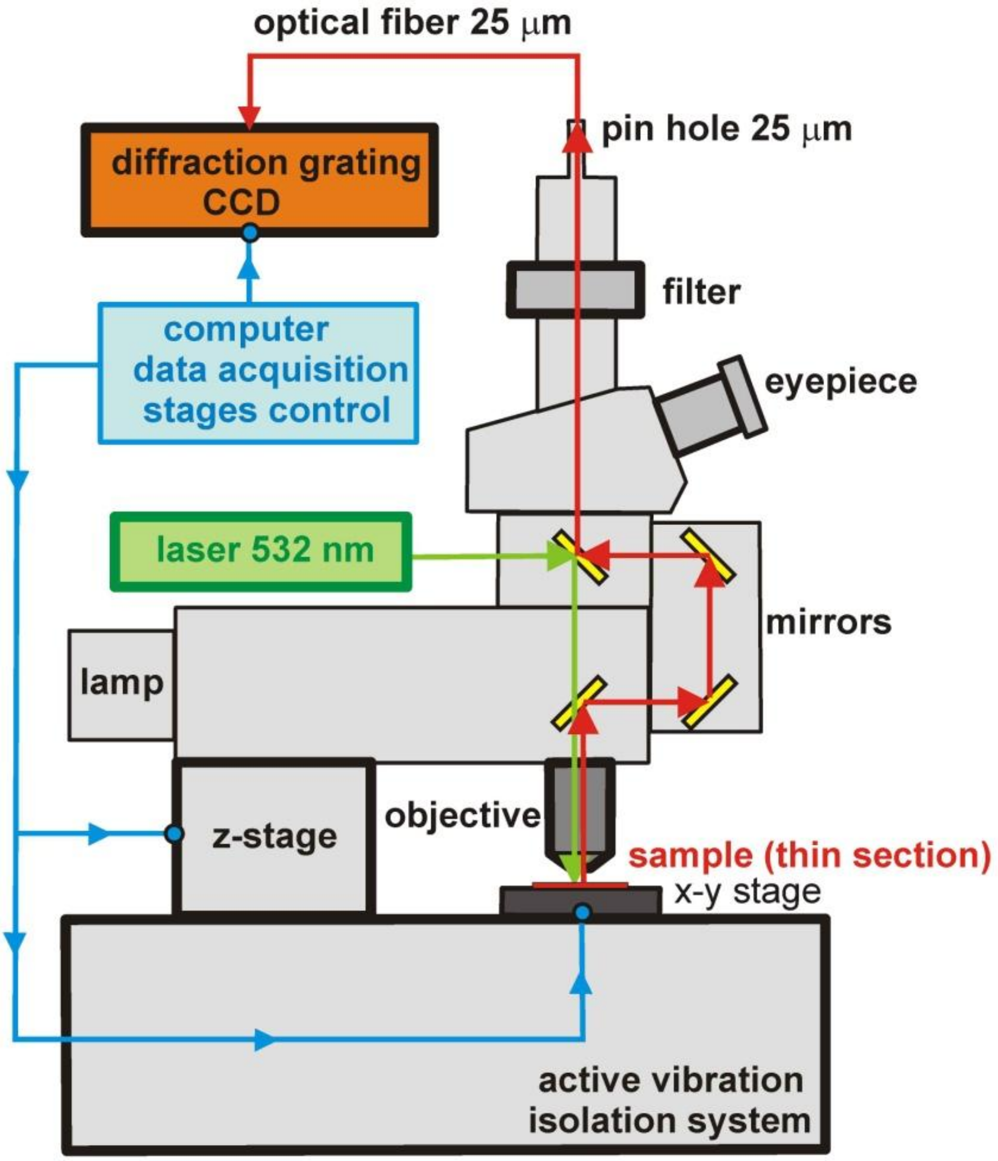

Supplementary Figure 3a

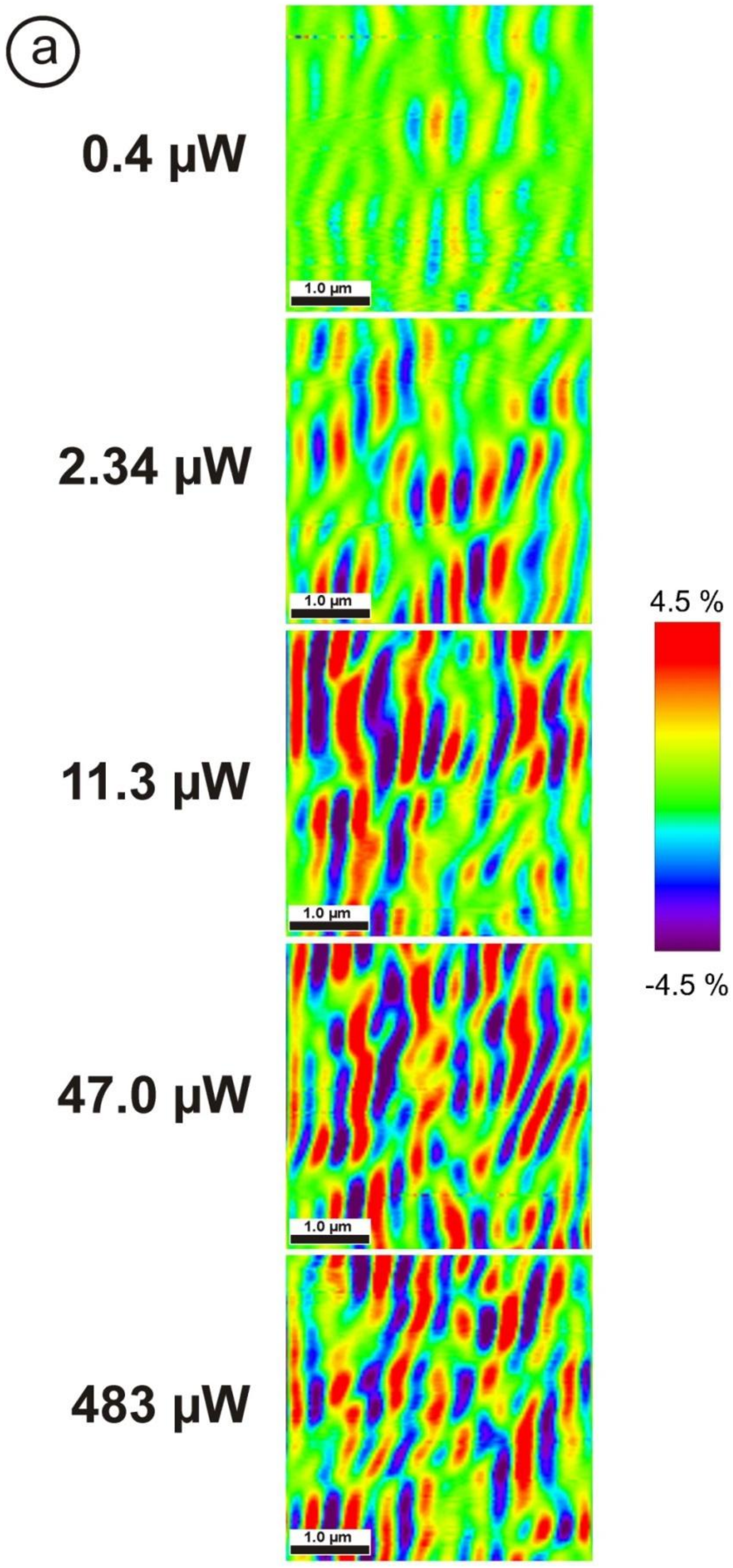

Supplementary Figure 3b

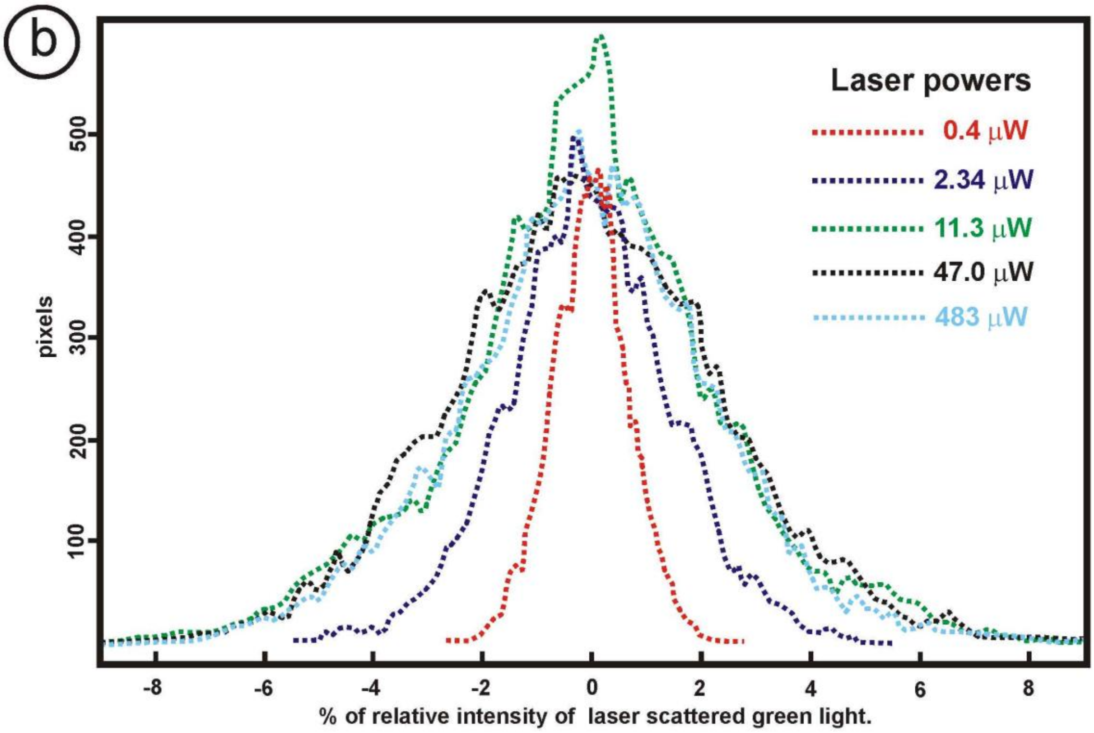

Supplementary Figure 4

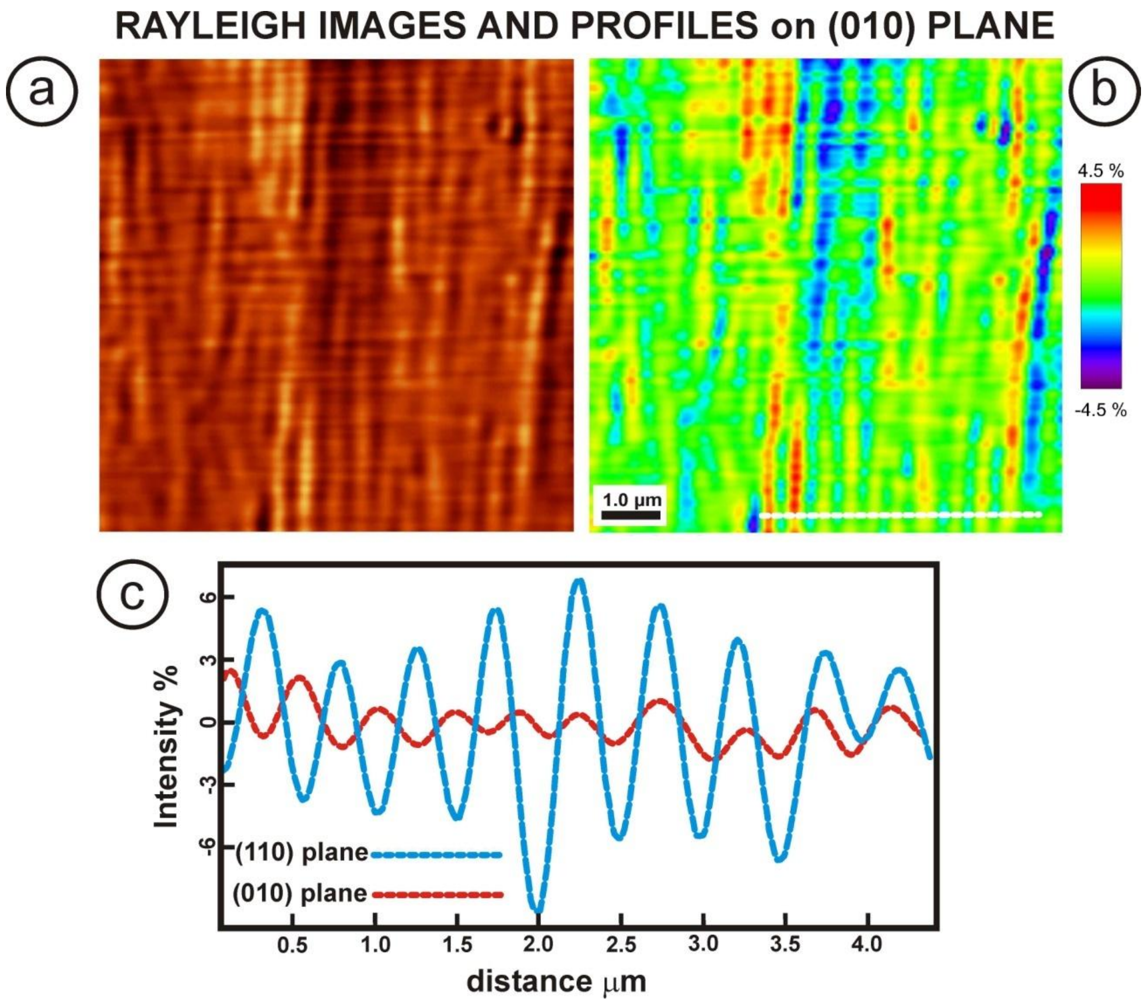

Supplementary Fig. S5

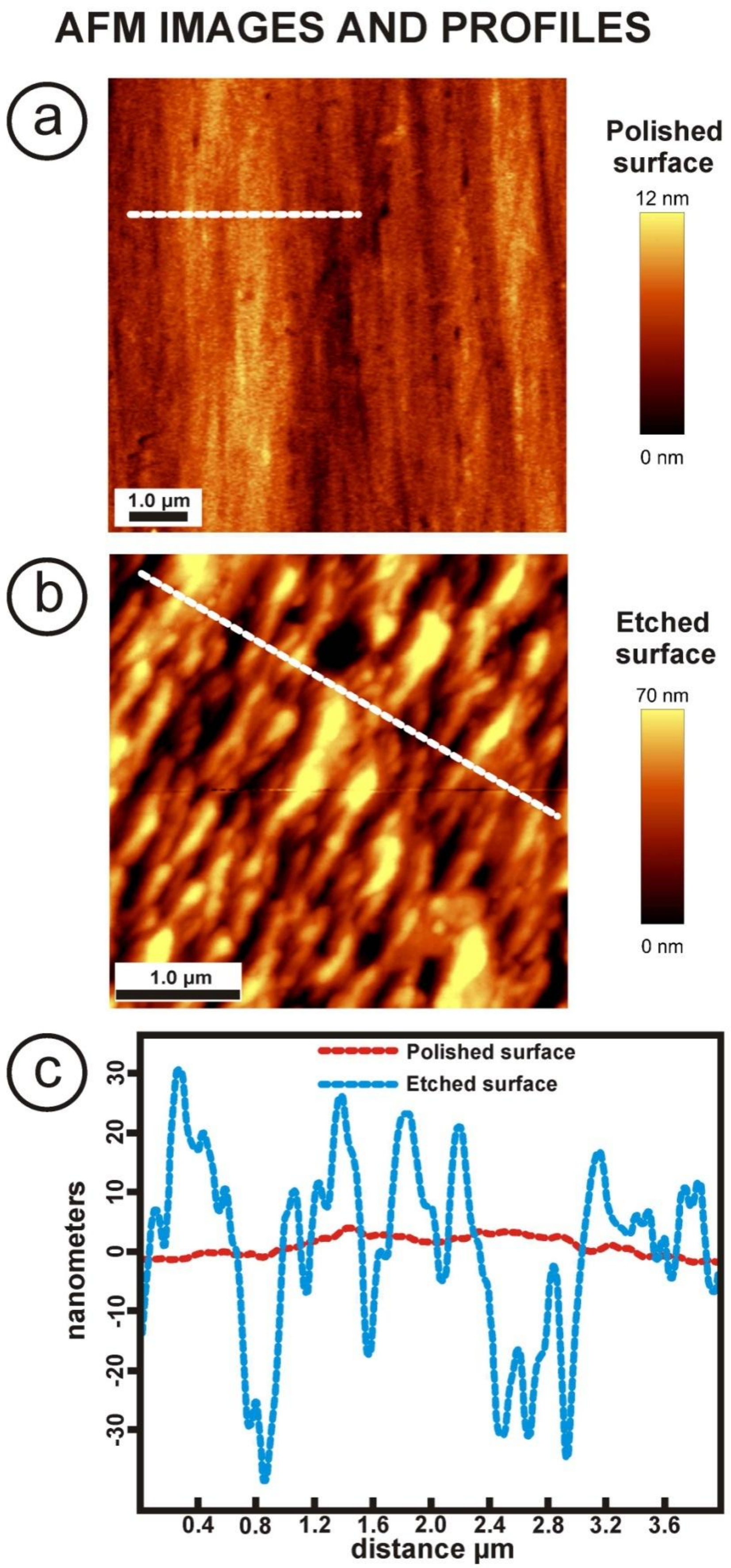

Supplementary Fig. S6

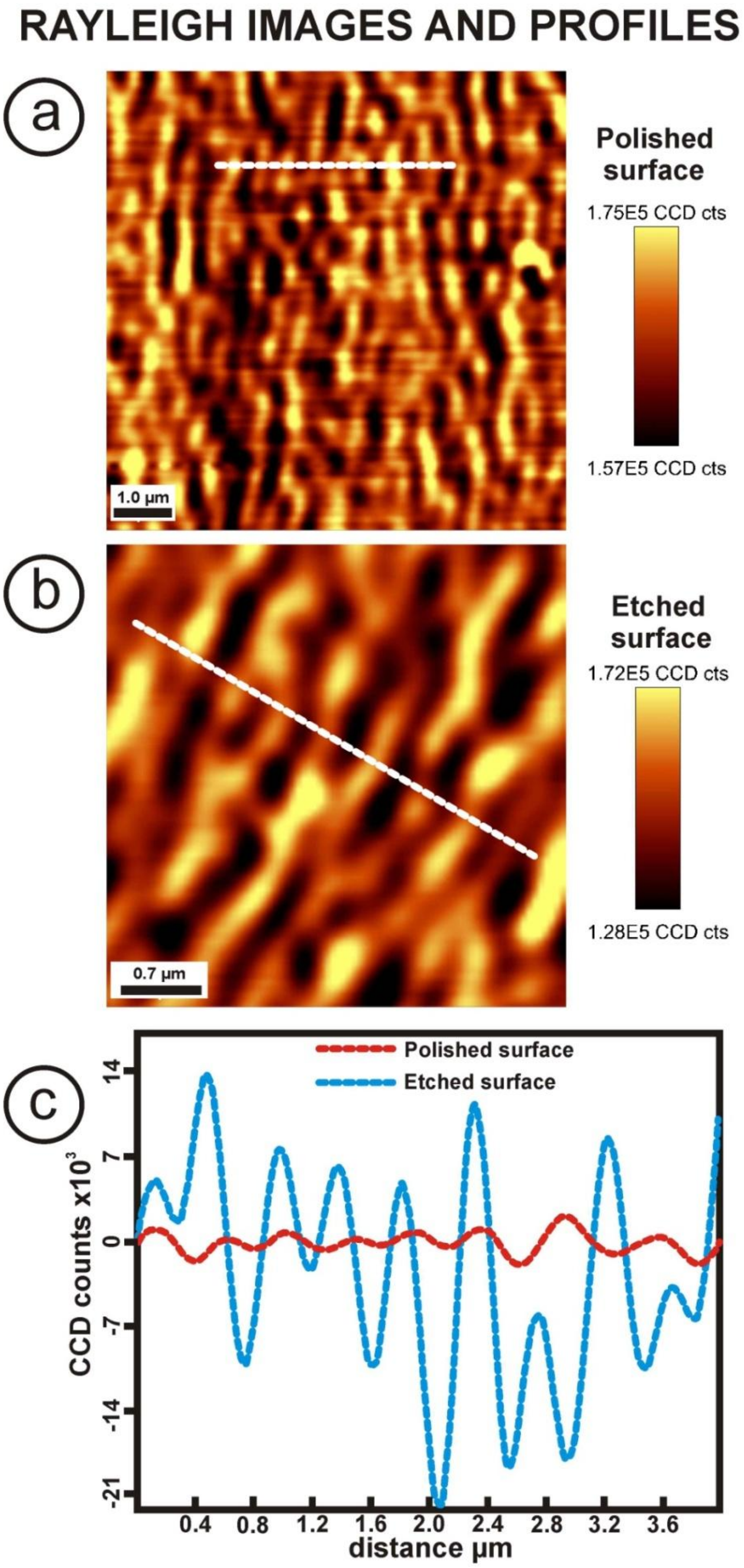

Supplementary Fig. S7

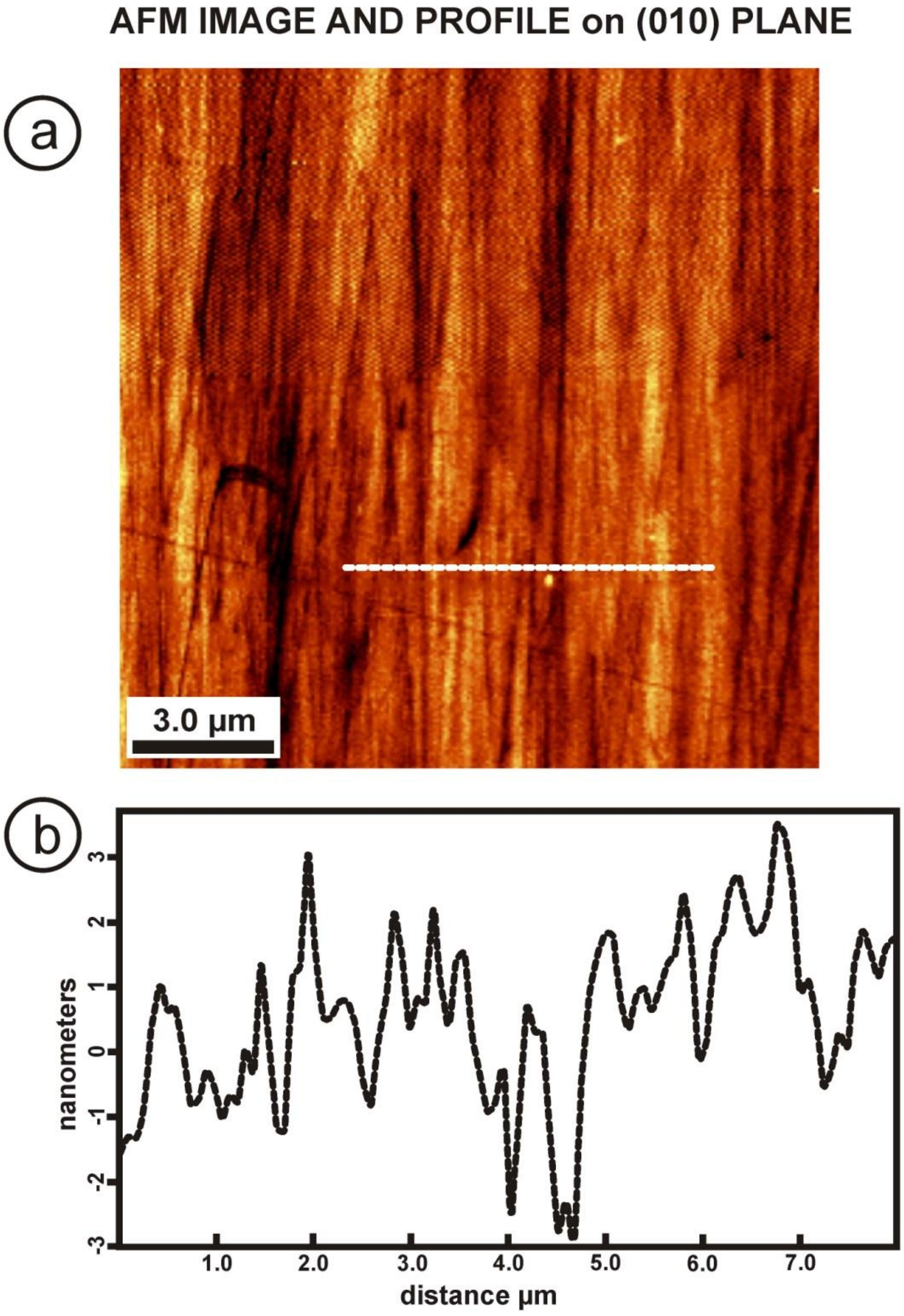

Supplementary Fig. S8

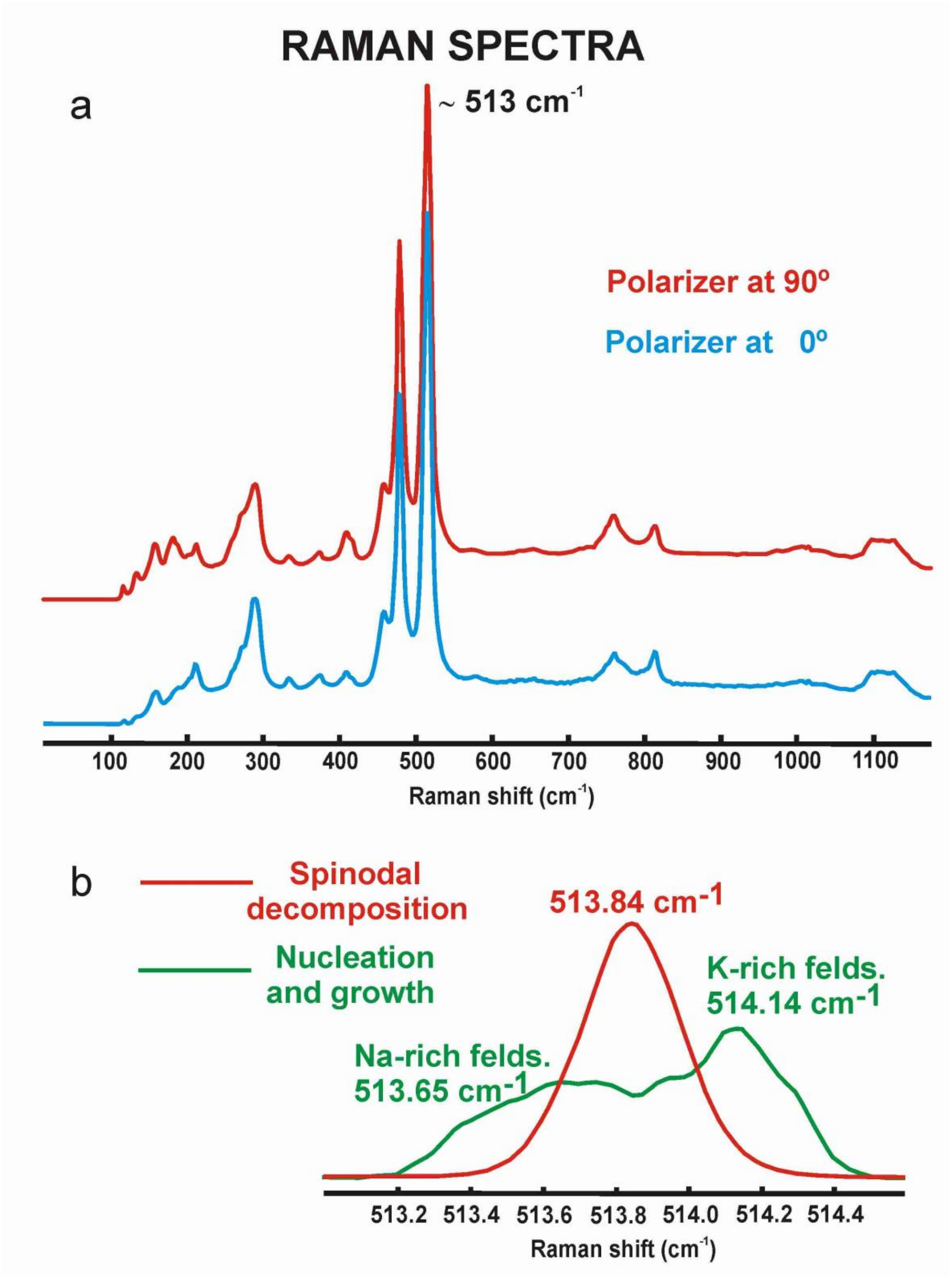

Supplementary Fig. S9

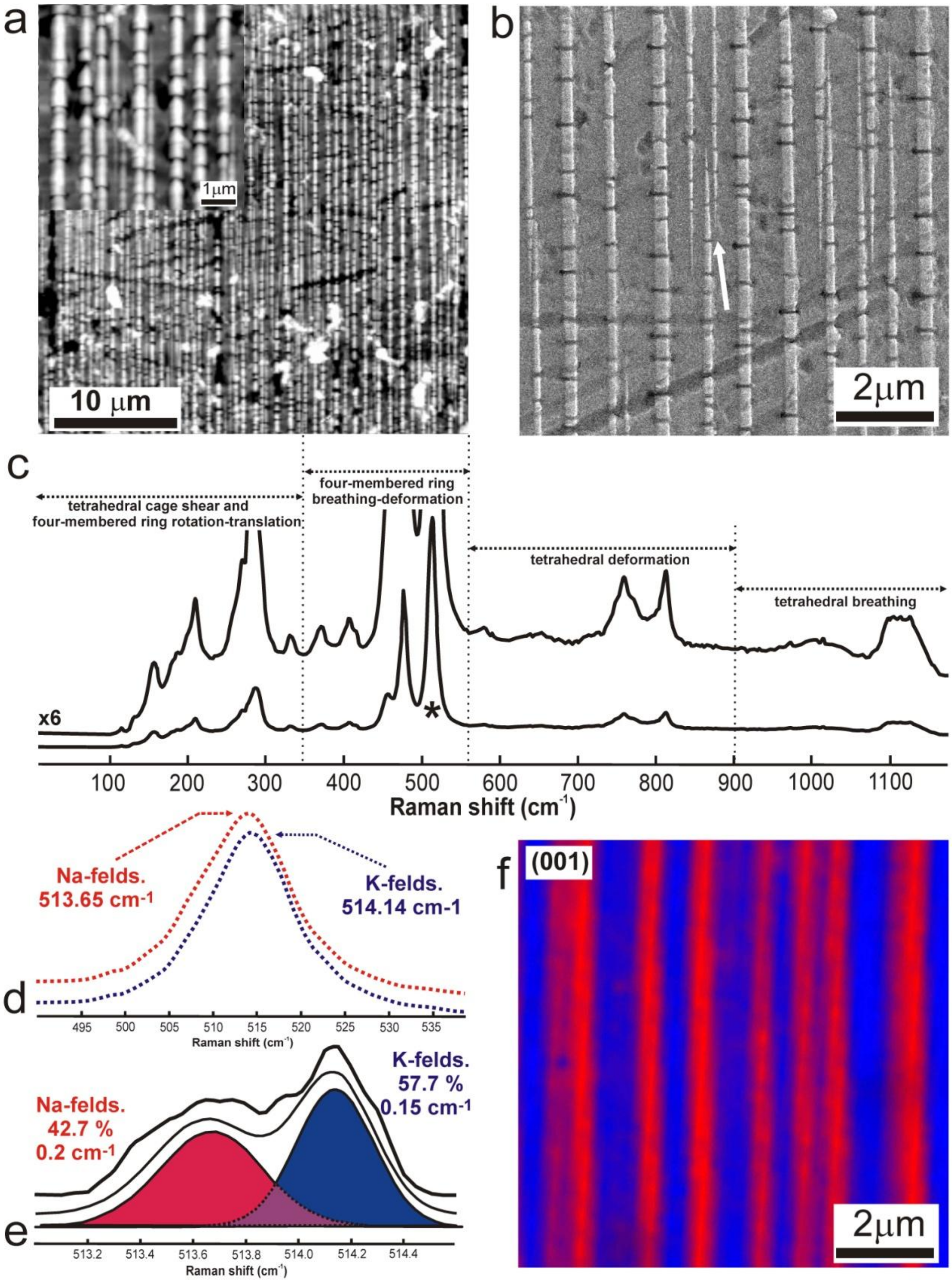

Supplement: Supplementary Information [file srep20806-s1.pdf]
